# Acute Ablation of Cortical Pericytes Leads to Rapid Neurovascular Uncoupling

**DOI:** 10.3389/fncel.2020.00027

**Published:** 2020-02-14

**Authors:** Kassandra Kisler, Angeliki M. Nikolakopoulou, Melanie D. Sweeney, Divna Lazic, Zhen Zhao, Berislav V. Zlokovic

**Affiliations:** ^1^Department of Physiology and Neuroscience, The Zilkha Neurogenetic Institute, Keck School of Medicine of the University of Southern California, Los Angeles, CA, United States; ^2^Department of Neurobiology, Institute for Biological Research, University of Belgrade, Belgrade, Serbia

**Keywords:** neurovascular coupling, acute pericyte ablation, cerebral blood flow, capillary, laser doppler flowmetry, intrinsic optical signal imaging, voltage-sensitive dye imaging

## Abstract

Pericytes are perivascular mural cells that enwrap brain capillaries and maintain blood-brain barrier (BBB) integrity. Most studies suggest that pericytes regulate cerebral blood flow (CBF) and oxygen delivery to activated brain structures, known as neurovascular coupling. While we have previously shown that congenital loss of pericytes leads over time to aberrant hemodynamic responses, the effects of acute global pericyte loss on neurovascular coupling have not been studied. To address this, we used our recently reported inducible pericyte-specific Cre mouse line crossed to iDTR mice carrying Cre-dependent human diphtheria toxin (DT) receptor, which upon DT treatment leads to acute pericyte ablation. As expected, DT led to rapid progressive loss of pericyte coverage of cortical capillaries up to 50% at 3 days post-DT, which correlated with approximately 50% reductions in stimulus-induced CBF responses measured with laser doppler flowmetry (LDF) and/or intrinsic optical signal (IOS) imaging. Endothelial response to acetylcholine, microvascular density, and neuronal evoked membrane potential responses remained, however, unchanged, as well as arteriolar smooth muscle cell (SMC) coverage and functional responses to adenosine, as we previously reported. Together, these data suggest that neurovascular uncoupling in this model is driven by pericyte loss, but not other vascular deficits or neuronal dysfunction. These results further support the role of pericytes in CBF regulation and may have implications for neurological conditions associated with rapid pericyte loss such as hypoperfusion and stroke, as well as conditions where the exact time course of global regional pericyte loss is less clear, such as Alzheimer’s disease (AD) and other neurogenerative disorders.

## Introduction

Proper brain functioning depends on delivery of oxygen and nutrients *via* cerebral blood flow (CBF). Neurovascular coupling, the unique mechanism of CBF control in the mammalian brain, ensures a rapid increase in the rate of CBF and oxygen delivery to activated brain structures (Kisler et al., 2017a). Pericytes are perivascular mural cells that enwrap brain capillaries. They are centrally positioned within the neurovascular unit, a collection of different cell types that at the level of brain capillaries includes pericytes, endothelial cells, astrocytes and neurons (Kisler et al., 2017a). Our group and others have shown that pericytes play vital roles in regulation of CBF (Bell et al., 2010; Tachibana et al., 2018; Nikolakopoulou et al., 2019; Nortley et al., 2019), neurovascular coupling (Peppiatt et al., 2006; Hall et al., 2014; Biesecker et al., 2016; Mishra et al., 2016; Kisler et al., 2017b; Cai et al., 2018; Rungta et al., 2018; Nortley et al., 2019), and blood-brain barrier (BBB) integrity (Armulik et al., 2010; Bell et al., 2010; Daneman et al., 2010).

Previously studied models of congenital pericyte deficiency rely on either reduced bioavailability of endothelial platelet-derived growth factor-B (PDGF-BB; Armulik et al., 2010; Keller et al., 2013) or globally inherited platelet-derived growth factor receptor-β (PDGFRβ) deficiency in pericytes (Bell et al., 2010; Daneman et al., 2010; Nikolakopoulou et al., 2017). *Pdgfrb*-deficient mice develop progressive but slow pericyte loss over time associated with CBF reductions and dysregulation, eventually leading to neuronal dysfunction as they age that may take months to develop (Bell et al., 2010; Kisler et al., 2017b; Montagne et al., 2018). While we have previously shown that moderate loss of pericytes in *Pdgfrb* pericyte-deficient mice leads to neurovascular uncoupling and diminished oxygen delivery preceding late-appearing neuronal changes (Kisler et al., 2017b), it is not clear if any developmental compensation occurred in this model that might contribute to aberrant neurovascular coupling. Using an *in vivo* laser ablation technique, another study has shown that acute single pericyte ablation leads to localized temporary loss of vascular tone and capillary dilation (Berthiaume et al., 2018). However, neither of these studies examined the effect of the rapid and global loss of brain pericytes on hemodynamic responses, as it may occur in some acute and chronic neurological disorders (Sweeney et al., 2018a,b, 2019).

To disentangle alterations in neurovascular coupling from developmental aspects of pericyte loss, and determine the effect of global acute pericyte loss on neurovascular coupling, we used a recently developed mouse model of pericyte-specific ablation to rapidly deplete pericytes from the brains of living mice (Nikolakopoulou et al., 2019). We hypothesized that rapid loss of capillary pericyte coverage would lead to rapid aberrant hemodynamic responses to neuronal stimulus.

## Materials and Methods

### Animals

Mice were housed in plastic cages on a 12 h light cycle with *ad libitum* access to water and a standard laboratory diet. All procedures were approved by the Institutional Animal Care and Use Committee at the University of Southern California with the National Institutes of Health guidelines. Animals of both sexes 2–3 months old were used in the experiments. All animals were randomized for their genotype information. All experiments were blinded: the operators responsible for the experimental procedures and data analysis were blinded and unaware of group allocation throughout the experiments.

Pericyte-CreER mice, which express Cre recombinase specifically in pericytes after induction with tamoxifen (TAM) treatment, were generated using a double-promoter approach combining a Pdgfrb-Flp construct that expresses Flp recombinase under the control of the Pdgfrb promoter (Foo et al., 2006; Cuttler et al., 2011) and a Cspg4-FSF-CreER construct carrying a Frt-Stop-Frt-CreER cassette under the control of Cspg4 promoter (Zhu et al., 2008, 2011), as we described previously (Nikolakopoulou et al., 2019). These mice were crossed with iDTR mice (Jackson Laboratory, Bar Harbor, ME, USA #: 007900) for Cre-dependent expression of diphtheria toxin (DT) receptor (DTR; from simian Hbegf; Buch et al., 2005) in pericytes. Tamoxifen (TAM) was administered intraperitoneally (i.p) to mice (40 mg/kg daily) for 7 days to induce DTR expression. Two weeks after the end of TAM treatment, 2–3 months old Pericyte-CreER; iDTR mice were administered i.p. 0.1 μg DT (Sigma–Aldrich, St. Louis, MO, USA #D0564) or vehicle per day for 10 consecutive days, as we reported previously (Nikolakopoulou et al., 2019). Animals were studied at day 0, 3, 6, and 9 of DT or vehicle treatment including laser doppler flowmetry (LDF), intrinsic optical signal (IOS) imaging, and pericyte coverage) and 3 days post-DT or vehicle (LDF, IOS imaging, pericyte coverage, neuronal response by voltage-sensitive dye (VSD) imaging, vascular density).

### Immunohistochemistry

Immunostaining was performed as described previously (Bell et al., 2010; Nikolakopoulou et al., 2017, 2019). Briefly, animals were anesthetized with an i.p. injection of 100 mg/kg of ketamine and 10 mg/kg of xylazine, and transcardially perfused first with 15 ml saline, followed by 20 ml of 4% paraformaldehyde (PFA) in PBS. Brains were removed and postfixed overnight with 4% PFA at 4°C before brain sections were cut at 30 μm thickness. The sections were blocked with 5% normal donkey serum (Vector Laboratories, Burlingame, CA, USA)/0.1%Triton-X/0.01M PBS and incubated with primary antibody for pericytes, polyclonal goat anti-mouse aminopeptidase N/ANPEP (CD13; R&D Systems, AF2335; 1:250), diluted in blocking solution overnight at 4°C. The sections were then washed in PBS and incubated with fluorophore-conjugated secondary antibody Alexa fluor 647-conjugated donkey anti-goat (Invitrogen, Waltham, MA, USA, A-21447, 1:500) for 1 h. To visualize brain microvessels, sections were incubated with Dylight 488-conjugated L. esculentum Lectin as we have previously reported (Bell et al., 2010; Nikolakopoulou et al., 2017) with the secondary antibodies. The sections were then washed in PBS and mounted onto slides with 4′,6-diamidino-2-phenylindole (DAPI) fluorescence mounting medium (Dako). The sections were imaged with a Zeiss LSM 510 confocal laser-scanning microscope, as we described previously (Montagne et al., 2018). Z-stack projections and pseudo-coloring were performed using ZEN software (Carl Zeiss Microimaging, Jena, Germany), and image post-analysis was performed using ImageJ software.

To determine pericyte coverage, 10 μm maximum projection z-stacks (area 640 × 480 μm) were reconstructed, and the areas occupied by CD13-positive (pericyte) and lectin-positive (endothelium) fluorescent signals on vessels <6 μm in diameter were analyzed using ImageJ as we described previously (Nikolakopoulou et al., 2017). For each animal, four to six randomly selected fields in the S1 somatosensory cortex region were analyzed in three to four non-adjacent sections (~100 μm apart) and averaged per mouse. Pericyte coverage of control mice that were not treated with DT was taken arbitrarily as zero loss of pericyte coverage (day 0).

To determine microvascular density, 10-micron maximum projection Z-stacks were reconstructed, and the length of lectin-positive capillary profiles (≤6 μm in diameter) were measured using the ImageJ plugin “Neuro J” length analysis tool. In each animal, 4–6 randomly selected fields (640 × 480 μm) in the somatosensory cortex were analyzed from four non-adjacent sections (~100 μm apart), and averaged per mouse, as we have previously described (Bell et al., 2010; Nikolakopoulou et al., 2017). The length was expressed in mm of lectin-positive vascular profiles per mm^3^ of brain tissue.

### Cranial Window

Cranial windows were implanted as previously described (Kisler et al., 2017b, 2018). Briefly, animals were initially anesthetized with 100 mg/kg of ketamine and 10 mg/kg of xylazine and were placed on a heating pad (37°C). The cranium of the mouse was firmly secured in a stereotaxic frame (Kopf Instruments, Tujunga, CA, USA). A high-speed dental drill (tip FST 19007-05, Fine Science Tools Inc., Foster City, CA, USA) was used to delineate a cranial window about 5 mm in diameter over the somatosensory cortex, and 45° forceps were used to remove the piece of skull. Gelfoam (Pharmacia and Upjohn Company, Kalamazoo, MA, USA) was applied immediately to control any cranial or dural bleeding. A sterile 5 mm glass coverslip was then placed on the dura mater and sealed with cyanoacrylate based glue.

### Laser-Doppler Flowmetry (LDF)

CBF responses to hind-limb stimulation in anesthetized mice (1% isoflurane) were determined using laser-Doppler flowmetry measured through a cranial window. The tip of the laser-Doppler probe (Transonic Systems Inc., Ithaca, NY, USA) was stereotaxically placed 0.5 mm above the cranial window. CBF was recorded from the somatosensory cortex hind-limb region following electrical stimulation of the hind-limb using a 60 s long stimulus (7 Hz, 2 ms pulse duration). The percent increase in CBF due to stimulation was obtained by subtracting the baseline CBF from the maximum value reached during stimulus, and averaged over three trials per mouse. For CBF response to acetylcholine application, the LDF probe was stereotaxically placed over the center of an open cranial window (center at AP = −1.5 mm, *L* = 2 mm). Acetylcholine (10 μM, Sigma–Aldrich, St. Louis, MO, USA) was superfused over the open window, and responses recorded, as we reported previously (Kisler et al., 2017b; Nikolakopoulou et al., 2019).

### Intrinsic Optical Signal Imaging (IOS)

IOSs were imaged through a cranial window over the hind-limb region in the somatosensory cortex, as we previously described (Kisler et al., 2017b, 2018). Under isoflurane anesthesia set at 1%, images were captured at 30 ms/frame using a 1/2 inch CCD MiCAM02-HR camera (SciMedia; 2× binned to 184 × 124-pixel resolution; 1 pixel = 16.5 μm) with accompanying BV_ANA acquisition software. Images were captured under a 530 nm green LED light source and collected through a 522/36 nm bandpass filter (Chroma). The contralateral hind-limb was stimulated by a brief mechanical vibration lasting 300 ms. The resulting image sets were low pass filtered at 2 Hz, and the baselines corrected for any drift using the BV_ANA software. Signal time courses were evaluated using 10-pixel radius ROIs chosen in the region of peak signal change such that the regions did not include any large visible vessels, and were at least 30 μm away from any large vessels. Time courses were plotted using Igor Pro 6 and analyzed with Igor Pro 6 and GraphPad Prism 8. Pseudocolor images for presentation were generated in ImageJ.

### Voltage-Sensitive Dye (VSD) Imaging

VSD imaging was performed as described previously (Kisler et al., 2017b). Briefly, a cranial window was created over the hind-limb region in the somatosensory cortex, as described above. RH-1692 VSD (1 mg/ml; Optical Imaging) dissolved in aCSF was applied to the exposed cortex for 90 min. The brain was then washed with aCSF and sealed with a coverslip as above. Under isoflurane anesthesia (1%), images were captured at 5 ms/frame (184 × 124 resolution) using a 1/2 inch CCD MiCAM02-HR camera (SciMedia, Costa Mesa, CA, USA) coupled with MiCAM BV_ANA acquisition software. RH-1692 was excited using an MHAB-150W (Moritex Corp.) light source with a 632/22 filter, and fluorescence collected with a 665 nm long-pass filter. The contralateral hind-limb was stimulated by a brief mechanical vibration lasting 300 ms. Alternating image sets were taken with and without stimulus to generate “stimulus trial” and “baseline” responses. Next, the baseline image set was subtracted from the stimulus trials to eliminate any background signal. Ten baseline subtracted trials were averaged to make up the final profile for each mouse. Time courses were evaluated using a circular ROI centered over the hind-limb region as described previously (Bell et al., 2012; Kisler et al., 2017b). Time courses were plotted using Igor Pro 6 and analyzed with Igor Pro 6 and GraphPad Prism 8. Pseudocolor images for presentation were generated in ImageJ.

### Statistical Analysis

Sample sizes were calculated using nQUERY assuming a two-sided alpha-level of 0.05, 80% power, and homogeneous variances for the samples to be compared, with the means and common standard deviation for different parameters estimated based on our previous studies (Kisler et al., 2017b; Nikolakopoulou et al., 2017, 2019; Montagne et al., 2018). Data are presented as mean ± SEM. For comparison between two groups, an *F*-test was conducted to determine the similarity in variances between the groups that are statistically compared, and statistical significance was analyzed by two-tailed student’s *t-test*. For multiple comparisons, Brown–Forsythe test was used to determine the similarity in variances between the groups, and one-way analysis of variance (ANOVA) followed by Bonferroni or Tukey’s *post hoc* test was used as indicated in figure legends to test statistical significance, using GraphPad Prism 8 software. A *P*-value of less than 0.05 was considered statistically significant. Pearson correlation coefficient and significance were calculated using a two-tailed test in GraphPad Prism 8 software.

## Results

**Figure 1:**
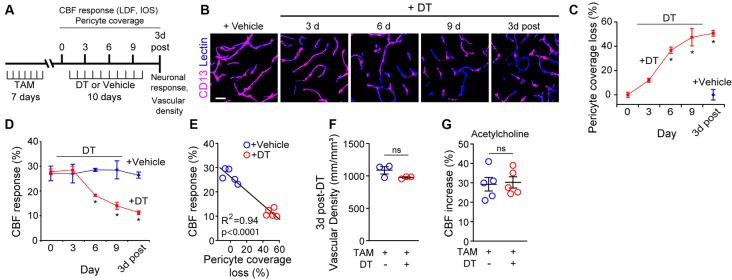
Ablation of cortical pericytes from the adult mouse brain leads to acute dysregulation of neurovascular coupling. **(A)** A diagram of the injection protocol of pericyte-CreER; iDTR mice with tamoxifen (TAM; 40 mg/kg daily for seven consecutive days), diphtheria toxin (DT; 0.1 μg per day for 10 consecutive days) or vehicle, and the time points when cerebral blood flow (CBF) responses to stimulus measured by laser Doppler flowmetry (LDF) and intrinsic optical signal (IOS) imaging, neuronal response to stimulus, and capillary density measurements were performed. **(B)** Representative confocal microscopy images of CD13-positive pericyte coverage of lectin-positive endothelial profiles in the S1 cortex hind-limb region at 3, 6, and 9 days of DT or vehicle administration, and 3 days post-DT or vehicle. Bar = 20 μm. **(C)** Quantification of pericyte coverage loss on capillaries (<6 μm in diameter) in the S1 cortex in TAM-treated pericyte-CreER; iDTR mice at 0, 3, 6 and 9 days of DT administration, and 3 days post-DT or vehicle treatment; *n* = 5 mice per group. *P < 0.05 vs. day 0 of DT treatment by analysis of variance (ANOVA) followed by Tukey’s *post hoc* test. **(D)** CBF response to an electrical hind limb stimulus (60 s duration, 7 Hz, 2 ms pulse duration) in 3-month-old TAM-treated pericyte-CreER; iDTR mice determined by LDF in the S1 cortex hind-limb region at 0, 3, 6, and 9 days of DT or vehicle administration, and 3 days post-DT or vehicle treatment. CBF response is expressed as the percentage increase relative to baseline; *n* = 5 mice per group; *P < 0.05, by ANOVA followed by Tukey’s *post hoc* test. **(E)** Pearson’s correlation between CBF response to a stimulus as in **(D)** and loss of pericyte coverage determined at 3 days post-DT or vehicle treatment of TAM-treated pericyte-CreER; iDTR mice. Each point represents an individual response per mouse of 10 studied mice; *P* < 0.0001. Significance by two-tailed Pearson correlation; R, Pearson correlation coefficient. **(F)** Capillary (diameter <6 μm) density in the S1 cortex hind-limb region in TAM-treated pericyte-CreER; iDTR mice at 3 days post-DT or vehicle treatment; *n* = 3 mice per group. **(G)** LDF measurements of CBF response to endothelium-dependent vasodilator acetylcholine (10 μM) in TAM-treated pericyte-CreER; iDTR mice determined 3 days post-DT or vehicle treatment; *n* = 5 mice per group. Data in **(C,D,F,G)** represented as Mean ± SEM; in **(F,G)** ns, non-significant by student’s *t*-test. Circles denote individual values per mouse in **(E–G)**.

To study the effect of acute and global pericyte loss on neurovascular coupling, we used our recently developed inducible pericyte-specific Cre mouse line (pericyte-CreER; Nikolakopoulou et al., 2019) crossed to iDTR mice carrying Cre-dependent human diphtheria toxin receptor (DTR; Buch et al., 2005; pericyte-CreER; iDTR), as we previously reported (Nikolakopoulou et al., 2019). Treatment of pericyte-CreER; iDTR mice with tamoxifen (TAM) induces DTR expression specifically in pericytes (and not in any other cell types), and subsequent treatment with DT leads exclusively to pericyte cell death, as previously reported (Nikolakopoulou et al., 2019). Deficits in neurovascular coupling were evaluated by LDF and IOS imaging of CBF responses to hindlimb stimulus at 0, 3, 6 and 9 days of DT or vehicle treatment, and 3 days post-DT or vehicle (Figure 1A). Pericyte coverage was determined at 0, 3, 6 and 9 days of DT treatment, and 3 days post-DT or vehicle, while VSD measurements of neuronal evoked membrane potential responses and cortical capillary density were evaluated at 3 days post-DT or vehicle treatment (Figure 1A).

As we reported (Nikolakopoulou et al., 2019), DT treatment of TAM-treated pericyte-CreER; iDTR mice compared to vehicle led to progressive loss of CD13-positive pericyte coverage (Figure 1B) as shown in the S1 somatosensory cortex by ~37% loss after 6 days of DT treatment, and by approximately 50% loss at 3 days post-DT treatment (Figure 1C) consistent with our previous report (Nikolakopoulou et al., 2019). Analysis of CBF responses to hind-limb stimulation as measured by LDF in the S1 cortex hind-limb region in TAM-treated pericyte-CreER; iDTR mice revealed progressive neurovascular dysregulation beginning with a 36% reduction in CBF response at day 6 of DT treatment compared to vehicle, and reaching 57% reduction in CBF response at 3 days post-DT compared to vehicle (Figure 1D). Diminished CBF responses in pericyte-CreER; iDTR mice treated with TAM and vehicle, or TAM and DT, correlated positively with the loss in pericyte coverage as shown at 3 days post-DT or vehicle treatment (Figure 1E).

The length of lectin-positive capillary (diameter <6 μm) profiles in the S1 cortex (Figures 1B,F) was unchanged in pericyte-CreER; iDTR mice at 3 days post-DT or vehicle treatment, indicating that the observed differences in CBF responses could not be attributed to differences in brain capillary density. To determine whether endothelial dysfunction can contribute to CBF dysregulation as seen in TAM-treated pericyte-CreER; iDTR mice after DT treatment, we tested CBF response to acetylcholine, an endothelium-dependent receptor-mediated vasodilator (Figure 1G; Kisler et al., 2017b). These experiments showed no difference in CBF response in the presence of acetylcholine at 3 days post-DT or vehicle treatment, thus ruling out endothelial dysfunction as a contributor to the observed changes in neurovascular coupling.

**Figure 2:**
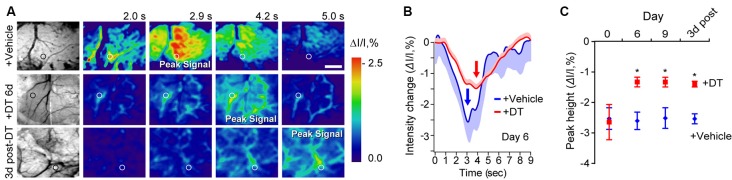
Neurovascular coupling deficits in pericyte-CreER; iDTR mice at key time points during pericyte ablation with DT and 3 days post-DT determined by IOS imaging. **(A)** Example of grayscale images of the visualized somatosensory cortex of TAM-treated pericyte-CreER; iDTR mice showing vasculature (far left), and pseudocolored images of the somatosensory cortex showing IOS imaging under 530 nm illumination in response to a 300 ms mechanical hind limb stimulus beginning at 0 s at key time points during DT (+DT) or vehicle administration. Peak signals and peak signal times are indicated. Circles indicate regions of interest (ROIs) for curves shown in panel **(B)** for day 6 of DT treatment. Scale bar = 0.5 mm. **(B)** IOS time courses for vehicle and 6 days DT-treated mice in parenchymal ROIs shown in **(A)**. Mean ± SEM from 10 trials in each representative mouse is shown. Arrows indicate peak signal intensity. **(C)** IOS peak intensity quantification at 0, 6, and 9 days of DT or vehicle administration, and 3 days post-DT or vehicle treatment in response to stimulus in ROIs away from large surface vessels in mice as in **(A)**. Mean ± SEM, *n* = 4–6 mice per group. *P < 0.05, one-way ANOVA followed by Bonferroni *post hoc* test.

To independently test whether DT-induced pericyte-deficiency can lead to a global deficit in neurovascular coupling in the parenchyma over the course of DT treatment, we studied hemodynamic responses to a mechanical hind-limb stimulus at key time points by IOS imaging acquired under 530 nm illumination (Figures 2A–C). IOS changes at this wavelength reflect changes in the total hemoglobin content in the brain tissue due to changes in blood flow and blood volume in response to stimulus (Hillman, 2007; Kisler et al., 2017b, 2018). At days 6 and 9 of DT treatment and 3 days post-DT treatment compared to vehicle, TAM-treated pericyte-CreER; iDTR mice showed a decrease in the peak signal IOS amplitude by approximately 50% starting at day 6 of DT treatment (Figure 2C), corroborating our LDF data by suggesting impaired neurovascular coupling immediately after pericyte loss.

**Figure 3:**
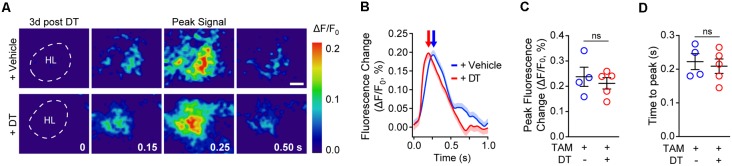
Cortical neuronal activity determined by voltage-sensitive dye (VSD) imaging in response to sensory stimulation in pericyte-CreER; iDTR mice 3 days post-DT treatment. **(A)** Representative pseudocolored image sequences of cortical depolarization pattern in the hind limb S1 cortical region obtained by VSD imaging in response to a 300 ms hind limb mechanical stimulus in TAM-treated pericyte-CreER; iDTR mice treated with DT or vehicle determined 3 days post-DT or vehicle treatment. The dashed line indicates hind limb (HL) region. Peak signals and peak signal times are indicated. Scale bar = 0.25 mm. **(B–D)** Representative VSD intensity traces from individual mice (average ± SEM for 10 trials per mouse; **B**), average peak fluorescence change **(C)**, and average time to peak **(D)** in pericyte-CreER; iDTR mice 3 days post-DT or vehicle treatment. In **(C**,**D)**, mean ± SEM, from *n* = 4–5 mice per group; circles denote individual values per mouse. ns, non-significant by student’s *t*-test.

Next, we investigated whether neurovascular coupling deficits could be due to changes in neuronal activation as evaluated by VSD imaging of neuronal evoked membrane potential responses to hind limb stimulus at 3 days post-DT or vehicle treatment. VSD imaging in the hind-limb S1 cortical area of TAM-treated pericyte-CreER; iDTR mice revealed no differences in depolarization pattern (Figures 3A–D), with similar signal amplitude (Figure 3C) and response latency (Figure 3D) at 3 days post-DT treatment compared to vehicle, consistent no changes in neuronal counts, neuritic density or behavior as previously reported in TAM-treated pericyte-CreER; iDTR mice at 3 days post-DT (Nikolakopoulou et al., 2019).

## Discussion

Using our recently developed pericyte-specific Cre line crossed to iDTR mice (Nikolakopoulou et al., 2019), we found that deficits in neurovascular coupling develop rapidly after global pericyte ablation from the cortex of adult mice. Furthermore, we also found that endothelial function and capillary density remain normal over the course of DT-induced pericyte loss, and that neuronal function evaluated by neuronal evoked membrane potential responses to hind limb stimulus was also unchanged 3 days after DT treatment in pericyte-CreER; iDTR mice. This data, together with our previous findings demonstrating unchanged arteriole smooth muscle cell (SMC) coverage and SMC functional response to adenosine after pericyte ablation with DT in TAM-treated pericyte-CreER; iDTR mice (Nikolakopoulou et al., 2019), suggests that acute loss of pericyte coverage, but not other vascular deficits or neuronal dysfunction, drives the observed deficits in neurovascular coupling.

We observe aberrant hemodynamic responses in TAM-treated pericyte-CreER; iDTR mice very early after an initial 37% loss of pericyte coverage occurring at 6 days of DT treatment. Multiple *in vivo* studies have indicated that pericytes regulate neurovascular coupling, playing an active role in capillary dilation (Hall et al., 2014; Biesecker et al., 2016; Mishra et al., 2016; Kisler et al., 2017b; Cai et al., 2018; Rungta et al., 2018; Nortley et al., 2019) that according to some studies precedes arteriolar dilation after neuronal stimulus (Hall et al., 2014; Mishra et al., 2016; Kisler et al., 2017b). Consistent with the present findings, recent work from our lab using a model of congenital pericyte deficiency due to globally inherited PDGFRβ deficiency in pericytes also revealed that loss of pericyte coverage delayed capillary vessel dilation upon neuronal stimulus (Kisler et al., 2017b). These studies suggest that acute pericyte loss may lead to aberrant hemodynamic responses due to a loss of active capillary dilation in response to neuronal stimulus. However, present literature also does not rule out a role for capillaries in the retrograde propagation of intramural vascular signals from the capillary level upstream influencing neurovascular coupling (Hillman, 2014; Longden et al., 2017), which would likely be compromised by the loss of pericytes and subsequent BBB breakdown, as discussed below.

A previous study found that significant resting-state CBF reductions and low but detectable BBB breakdown also develop after 6 days of DT treatment of TAM-treated pericyte-CreER; iDTR mice (Nikolakopoulou et al., 2019). Vasogenic edema, likely caused by a substantial BBB breakdown after 9 days of DT treatment (Nikolakopoulou et al., 2019), cannot be ruled out as a contributor to aberrant neurovascular coupling observed at later time points during and after the DT treatment regimen. Nonetheless, that we observe a neurovascular coupling deficit at a time point prior to edema development implies also that loss of pericyte coverage may be sufficient to initially drive changes in neurovascular coupling. Since our previous report found no changes in the number of Iba1-positive microglia and GFAP-positive astrocytes after pericyte ablation in the presently crossed pericyte-CreER; iDTR line (Nikolakopoulou et al., 2019), it is unlikely that the changes we observe in neurovascular coupling after pericyte loss are influenced by an inflammatory response. Given the apparent role that pericytes play in regulating the dynamic changes in hemodynamic responses (Hall et al., 2014; Biesecker et al., 2016; Mishra et al., 2016; Kisler et al., 2017b; Cai et al., 2018; Rungta et al., 2018; Nortley et al., 2019), it is possible that deficits in neurovascular coupling observed in the present study might also contribute to CBF reductions reported previously in this model (Nikolakopoulou et al., 2019).

A previous study investigating acute ablation of individual pericytes from the adult mouse brain found temporary loss of vascular tone and capillary dilation, but also revealed that neighboring pericytes possess the plasticity to extend processes into the region vacated by the ablated cell to restore pericyte coverage and tone of the affected capillary (Berthiaume et al., 2018). The effect of blood flow after single pericyte ablation in this model was not evaluated, but it is reasonable to assume that single pericyte loss likely would not cause large alterations to CBF. While single pericyte ablation technique yields important insights into the plasticity of pericytes and their regulation of resting vascular tone, it is difficult to directly compare these previous findings to the conditions presented here where brain-wide pericyte loss is rapidly induced. Such rapid, large scale pericyte loss as we observe in the present pericyte-CreER; iDTR model likely does not allow for sufficient numbers of pericytes to effectively compensate for lost pericyte coverage.

Rapid global pericyte loss from central nervous system occurs after cerebral hypoperfusion, stroke, and spinal cord injury (Melgar et al., 2005; Göritz et al., 2011; Fernández-Klett et al., 2013; Dias et al., 2018; Liu et al., 2019). While it is thought that some of these pericytes can detach from capillaries to allow neovascularization (Shimauchi-Ohtaki et al., 2019), contribute to scar formation (Göritz et al., 2011; Dias et al., 2018), or transform cell phenotype through their multipotent properties (Dore-Duffy, 2008; Nakagomi et al., 2015), the effects of rapid global pericyte loss on neurovascular coupling has still not been examined prior to the present study. Substantial regional pericyte loss (≥50%) in cortex and hippocampus is also observed in neurodegenerative diseases such as Alzheimer’s disease (AD; Sengillo et al., 2013; Halliday et al., 2016), but whether it occurs rapidly or more slowly over time is less clear. Aberrant neurovascular coupling and CBF reductions have been reported in humans with dementia, AD and other neurodegenerative diseases (reviewed in Iadecola, 2017; Sweeney et al., 2018a,b), and more recent studies suggest that pericyte injury is an early independent biomarker of human cognitive dysfunction (Montagne et al., 2015; Nation et al., 2019). Thus, the present findings, in addition to further supporting the role of pericytes in CBF regulation, may also have implications for understanding neurovascular dysfunction in disorders associated with global and rapid pericyte loss such as stroke, as well as conditions where the exact time course of global regional pericyte loss is less clear, such as AD and other neurogenerative disorders.

## Data Availability Statement

The data that support the findings of this study are available from the corresponding author upon reasonable request.

## Ethics Statement

All procedures performed on animals were reviewed and approved by the Institutional Animal Care and Use Committee at the University of Southern California.

## Author Contributions

KK, AN, ZZ, and BZ contributed to the project and experimental design. KK, AN, and MS conducted experiments and analyzed data. DL performed experiments. BZ supervised experiments. KK and BZ wrote the manuscript.

## Conflict of Interest

The authors declare that the research was conducted in the absence of any commercial or financial relationships that could be construed as a potential conflict of interest.
