# Corrigendum: Acute ablation of cortical pericytes leads to rapid neurovascular uncoupling

**DOI:** 10.3389/fncel.2022.1078919

**Published:** 2022-11-29

**Authors:** Kassandra Kisler, Angeliki M. Nikolakopoulou, Melanie D. Sweeney, Divna Lazic, Zhen Zhao, Berislav V. Zlokovic

**Affiliations:** ^1^Department of Physiology and Neuroscience, The Zilkha Neurogenetic Institute, Keck School of Medicine of the University of Southern California, Los Angeles, CA, United States; ^2^Department of Neurobiology, Institute for Biological Research, University of Belgrade, Belgrade, Serbia

**Keywords:** neurovascular coupling, acute pericyte ablation, cerebral blood flow, capillary, laser doppler flowmetry, intrinsic optical signal imaging, voltage-sensitive dye imaging

In the original article, there was an error in the representative image in Figure 1B panel “9 d” as published. A representative image of a tissue section from a pericyte-CreER-iDTR mouse treated for 6 days with DT (6 d) was placed incorrectly into the 9 day (9 d) representative image location.

**Figure 1:**
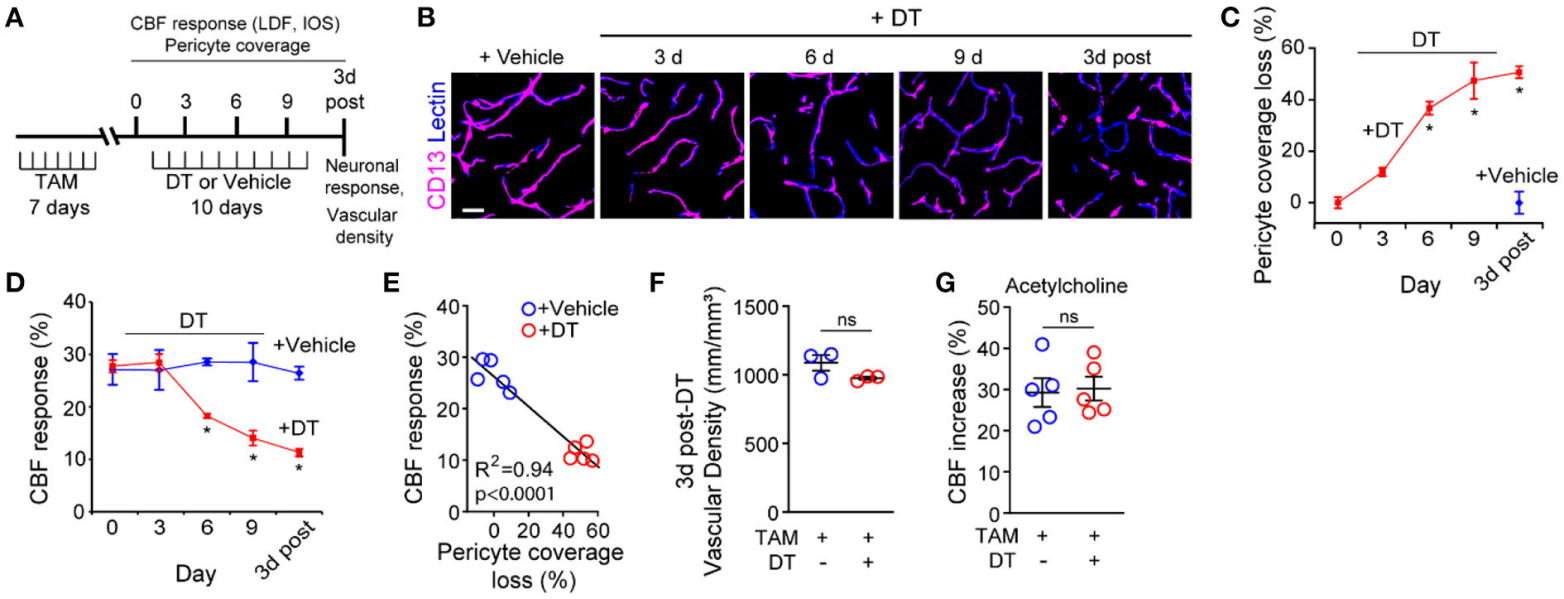
Ablation of cortical pericytes from the adult mouse brain leads to acute dysregulation of neurovascular coupling. **(A)** A diagram of the injection protocol of pericyte-CreER; iDTR mice with tamoxifen (TAM; 40 mg/kg daily for seven consecutive days), diphtheria toxin (DT; 0.1 mg per day for 10 consecutive days) or vehicle, and the time points when cerebral blood flow (CBF) responses to stimulus measured by laser Doppler flowmetry (LDF) and intrinsic optical signal (IOS) imaging, neuronal response to stimulus, and capillary density measurements were performed. **(B)** Representative confocal microscopy images of CD13-positive pericyte coverage of lectin-positive endothelial profiles in the S1 cortex hind-limb region at 3, 6, and 9 days of DT or vehicle administration, and 3 days post-DT or vehicle. Bar = 20 μm. **(C)** Quantification of pericyte coverage loss on capillaries (<6 μm in diameter) in the S1 cortex in TAM-treated pericyte-CreER; iDTR mice at 0, 3, 6, and 9 days of DT administration, and 3 days post-DT or vehicle treatment; *n* = 5 mice per group. ^*^P < 0.05 vs. day 0 of DT treatment by analysis of variance (ANOVA) followed by Tukey's *post hoc* test. **(D)** CBF response to an electrical hind limb stimulus (60 s duration, 7 Hz, 2 ms pulse duration) in 3-month-old TAM-treated pericyte-CreER; iDTR mice determined by LDF in the S1 cortex hind-limb region at 0, 3, 6, and 9 days of DT or vehicle administration, and 3 days post-DT or vehicle treatment. CBF response is expressed as the percentage increase relative to baseline; *n* = 5 mice per group; ^*^P < 0.05, by ANOVA followed by Tukey's *post hoc* test. **(E)** Pearson's correlation between CBF response to a stimulus as in **(D)** and loss of pericyte coverage determined at 3 days post-DT or vehicle treatment of TAM-treated pericyte-CreER; iDTR mice. Each point represents an individual response per mouse of 10 studied mice; *P* < 0.0001. Significance by two-tailed Pearson correlation; R, Pearson correlation coefficient. **(F)** Capillary (diameter <6 μm) density in the S1 cortex hind-limb region in TAM-treated pericyte-CreER; iDTR mice at 3 days post-DT or vehicle treatment; *n* = 3 mice per group. **(G)** LDF measurements of CBF response to endothelium-dependent vasodilator acetylcholine (10 μM) in TAM-treated pericyte-CreER; iDTR mice determined 3 days post-DT or vehicle treatment; *n* = 5 mice per group. Data in **(C,D,F,G)** represented as Mean ± SEM; in **(F,G)** ns, non-significant by Student's *t*-test. Circles denote individual values per mouse in **(E–G)**.

The corrected Figure 1 and its caption is shown below.

The authors apologize for this error and state that it does not change scientific conclusions of the article in any way. The original article has been updated.

## Publisher's note

All claims expressed in this article are solely those of the authors and do not necessarily represent those of their affiliated organizations, or those of the publisher, the editors and the reviewers. Any product that may be evaluated in this article, or claim that may be made by its manufacturer, is not guaranteed or endorsed by the publisher.

